# Quality assessment of prenatal and puerperium care

**DOI:** 10.31744/einstein_journal/2023AO0094

**Published:** 2023-07-25

**Authors:** Marcilene de Paula, Doroteia Aparecida Höfelmann

**Affiliations:** 1 Universidade Federal do Paraná Curitiba PR Brazil Universidade Federal do Paraná, Curitiba, PR, Brazil.

**Keywords:** Prenatal care, Quality of health care, Outcome and process assessment, health care, Family nurse practitioners, Public health

## Abstract

**Objective:**

To evaluate prenatal and puerperium care levels received and identify their association with sociodemographic and obstetric characteristics.

**Methods:**

This cross-sectional study was conducted from May to December 2020 and included women who gave birth at the Municipal Hospital of Fazenda Rio Grande, Paraná, Brazil. Data were collected through interviews and review of portfolios and medical records. The variables extracted from the prenatal protocols of Paraná and the Ministry of Health were grouped into five compliance indices: CI1 - clinical examination; CI2 - health education; CI3 - queries; CI4 - examinations and vaccines; and CI5 - postpartum appointments. Prenatal care was considered adequate when 80% or more adequacy was obtained.

**Results:**

A total of 307 women participated in this study. Prenatal compliance was 16.6% considering the entire set of variables. The best performance was for CI4 (54.7%) and the worst for CI5 (13.3%). The lowest adequacy occurred among single women (10.9%) compared to those who lived with a partner (19.9%) (p=0.043) and among women with black/brown skin color (9.5%) compared to those with white/yellow skin color (20.3%) (p=0.016).

**Conclusion:**

Most women did not receive adequate care, with those in situations of greater social vulnerability received worse quality care.

**Figure:**
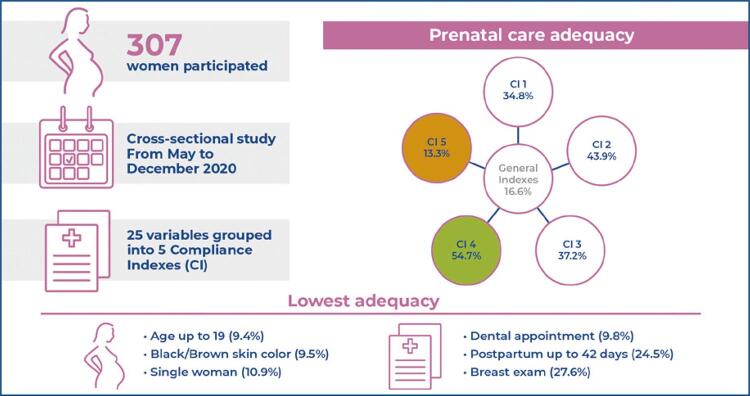

## INTRODUCTION

The goal of prenatal care is to avoid preventable maternal complications and ensure the birth of a healthy child. Most egalitarian societies, and those with greater investments in prenatal care, have better indicators of maternal and child health; on the other hand, countries with a greater degree of inequality have worse indicators.^( 1 )^

In Brazil, of the maternal deaths registered in the Mortality Information System (SIM - *Sistema de Informação sobre Mortalidade* ) from 1996 to 2018, approximately two thirds occurred due to direct obstetric causes, that is, obstetric complications during pregnancy, childbirth or puerperium caused by interventions, omissions, or incorrect treatment. Adequate care during pregnancy could reduce the number of these deaths, considering that most are preventable, such as hemorrhages and hypertensive disorders, which are the main causes of death during pregnancy, childbirth, and puerperium.^( 2 , 3 )^

Maternal mortality can be considered a social phenomenon because it is related to a population’s living conditions and access to qualified healthcare services, and no homogeneous distribution of mortality has been observed among Brazilian regions.^( 4 - 9 )^ In addition, individual differences suggest a pattern of mortality related to socioeconomic conditions, with worse rates among black or brown women and those with limited education.^( 4 - 9 )^The Birth in Brazil Survey reinforced the differences in the adequacy of prenatal care in public services, revealing inequality in access to and quality of prenatal care among Brazilian regions.^( 10 )^

To assess the quality of prenatal care, different indices have been proposed, with measurement methods focused on quantitative aspects, such as the number of appointments and gestational age at the beginning of prenatal care.^( 11 , 12 )^ However, these aspects are insufficient to broadly characterize the quality of prenatal care. Therefore, it is necessary to consider that the assessment process should be more comprehensive, including issues related to the content of the care provided in each contact between the pregnant woman and the healthcare service, such as educational activities, clinical actions, and gestational risk stratification, among others.^( 13 - 15 )^

## OBJECTIVE

To assess compliance with prenatal and postpartum care based on a general Compliance Index and identify associations with the sociodemographic, obstetric, and care characteristics of the women.

## METHODS

This cross-sectional study included women who gave birth at the *Hospital e Maternidade Nossa Senhora Aparecida* in the municipality of Fazenda Rio Grande, Paraná between May and December 2020. This municipality is a part of the Metropolitan Region of Curitiba and has a population of 100,209.^( 16 )^ The female population of childbearing age (10–49 years) represented 34.1% of the total population in 2010, the last Demographic Census.^( 17 )^ In 2015, the infant mortality rate was 19.1 deaths per 1000 births, with a reduction to 5.5/1000 in 2020.^( 18 )^

Regarding population coverage, in June 2020, the municipality had 16 accredited Family Health Teams, with a coverage percentage of 55.0%; in terms of attention to oral health, the coverage was 35.6% during the same period.^( 18 )^

The municipality has a maternity hospital financed exclusively by the Health Unic System (SUS *- Sistema Único de Saúde* ) and under municipal management. Data collection took place at this establishment. This is a general hospital with 32 active beds. Within the Guidelines of the Maternal and Child Network of the State of Paraná, it is a resource for all pregnant women in the municipality stratified as Habitual Risk,^( 2 )^ but considering that the municipality is far from a high-risk maternity hospital, this facility also provides emergency obstetric care for pregnant women with any degree of risk.

For sample estimation, 90 births per month in the maternity hospital were estimated, which totals 1,080 births per year. Considering the unknown prevalence of the outcome and prenatal adequacy, and to generate a larger sample size, we adopted an outcome prevalence of 50%, a margin of error of 5 percentage points, and a confidence level of 95%, totaling 284 pregnant women. Considering the possible losses and/or refusals of women to participate, 20% were added, totaling 341 pregnant women invited to participate in the study. The estimates were performed using OpenEpi version 3 a free and open code software developed by Dean, Sullivan & Soe (Georgia, United States).

The study population consisted of women in the puerperium period, enrolled before hospital discharge, who agreed to participate in the study, and whose delivery took place at the hospital in Fazenda Rio Grande from May to December 2020. Exclusion criteria were women who had received prenatal care totally or partially in the private network, who had received prenatal care totally or partially in other cities, or who did not agree to participate in the study. The included women were registered in an online electronic spreadsheet for later drawing.

Prenatal care data were extracted from pregnant women’s health records, and hospital data were extracted from patient records. Before hospital discharge, women were asked to complete a questionnaire that included socioeconomic data and prenatal care received. Forty-two days after delivery, the researcher contacted the participants to collect puerperal care data. This contact was made by phone or message exchange according to the preference the woman had initially indicated.

The variables selected for the study are included in the prenatal protocol of the Ministry of Health^( 19 )^ and Guideline of the Maternal and Child Network of Paraná.^( 2 )^ We assessed 25 variables grouped into five Compliance Indexes (CI): CI1 - cinical examination: Breast exam; measurement of uterine height; measurement of blood pressure; preventive screening examination for cervical cancer and gynecological examination; CI2 - health education: guidance on diet and weight gain, breast-feeding, childcare, the importance of preventive screening for cervical cancer, and the risks and benefits of each mode of delivery; CI3 - queries: prenatal care began in the first 12 weeks of pregnancy, gestational risk stratification at all appointments, risk stratification consistent with the Mother-Child Network Guideline, six or more prenatal appointments, undergoing a dental examination; CI4 - examinations and vaccines: and vaccines: attending the first routine examination, attending the second routine examination, undergoing the third set of routine laboratory tests, undergoing at least one ultrasound by the SUS, undergoing a rapid syphilis test; undergoing a rapid HIV test, tetanus vaccine and hepatitis B vaccine administration; CI5 - postpartum appointments: first postpartum appointment performed within 10 days after delivery, and second puerperium appointment performed within 42 days after delivery.^( 2 , 19 )^

The variables were grouped according to their similarity with the composition of each index. Each variable was classified in relation to the adequacy of the item, and the percentage of the adequacy of each index and the contribution of each variable to the overall compliance index were subsequently calculated.

To determine the association between the general compliance index and the characteristics of the women, the following variables were collected: age, skin color, per capita income, years of education, marital coexistence, paid occupation, parity, pregnancy planning, and satisfaction (with professionals and the team).

Continuous variables were described by calculating means, standard deviations, and minimum and maximum values. Absolute (n) and relative (%) frequencies were estimated for categorical variables.

The outcome was an overall compliance rate greater than or equal to 80%. This adequacy percentage reflects the level considered adequate by Kotelchuk’s APNCU Index (Adequacy of Prenatal Care Utilization).^( 12 )^ Associations between the investigated demographic, socioeconomic, and obstetric variables and the general index were tested using Pearson’s χ^2^ test and the linear trend test for ordinal categorical variables. Analyses were performed using Stata version 14.

Participants signed the Free and Informed Consent Term (TCLE), and those under the age of 18 signed the Free and Informed Assent Term while their legal guardians signed the TCLE. This study was approved by the Research Ethics Committee of the *Universidade Federal do Paraná* (CAAE: 78771317.2.0000.0102; # 4045.139).

## RESULTS

A total of 341 women were invited to participate in the study, of which 12 did not agree to participate, 13 dropped out in the second stage, and nine were excluded because they did not receive all their prenatal care at our facility, leaving 307 women who participated in the study.

Most women were between ages 20 and 29 years (60.6%), were white or yellow (65.8%), had a family income of less than BRL 456.00 monthly (67.8%), had ten years or more of education (85.3%), and lived with a partner (64.1%); 50.9% reported paid employment, 40.1% planned their pregnancy, and 32.2% were in their first pregnancy.

Prenatal care was considered adequate for 16.6% of the women. The index with the lowest compliance was CI5 (postpartum appointment), with 13.3% compliance, and the index with the best adequacy was CI4 (tests and vaccines), at 54.7%. A total of 0.3% of the women received all the care described in the set of variables. Among all variables, the lowest compliance item was having a dental appointment (9.8%). Table 1 presents the five compliance indices and the variables evaluated to compose the overall compliance index.

**Table 1:** Prenatal compliance rates in pregnant women included in the prenatal quality assessment survey

Index	Compliance (%)
Compliance Index 1	34.8
Breast exam	27.6
Uterine height measurement	97.7
Blood pressure measurement	97.0
Preventive collection	32.2
Gynecological examination	52.6
Compliance Index 2	43.9
Food and weight gain	74.0
Breastfeeding	64.5
RN care	55.3
Importance of preventive care	57.6
Delivery routes	45.7
Compliance Index 3	37.2
Beginning PN within 12 weeks	71.7
Attending six or more PN appointments	88.6
Risk stratification at all appointments	68.5
Risk stratification in accordance with published guidelines^( 2 )^	70.2
Attending a dental appointment	9.8
Compliance Index 4	54.7
Laboratory tests (three routines)	80.1
At least one ultrasound by SUS	80.1
HIV rapid test	73.0
Syphilis quick test	72.0
Tetanus vaccine	84.5
Hepatitis-B vaccine	64.3
Compliance Index 5	13.3
Postpartum appointment up to 10 days	40.1
Postpartum appointment up to 42 days	24.5
General Compliance Index	16.6

RN: newborn; PN: prenatal care; SUS: *Sistema Único de Saúde* .

The adequacy of prenatal care increases with the age of pregnant women. Less adequacy was found among those who did not live with a partner (10.9%) compared to those who lived with a partner (19.9%), and among women with black or brown skin color (9.5%) compared to those with white or yellow (20.3%) skin. The adequacy of prenatal care increased with the age of the pregnant women and decreased among black women (p=0.016) and those who did not live with a partner (p=0.043). The adequacy of prenatal care was greater among women who reported being satisfied with the professionals and the team, who would not change their basic health unit, and who would recommend it to friends and family members (p<0.01) ( Table 2 ).

**Table 2:** Associations between general index of compliance and sociodemographic, obstetric profile, and satisfaction with the team and professionals in pregnant women

Variables	Inappropriate prenatal care n (%)	Appropriate prenatal care n (%)	p value*
Age group (years)			0.018
Up to 19	29 (90.6)	3 (9.4)	0.006^†^
20–29	161 (86.6)	25 (13.4)	
30 or more	66 (74.2)	23 (25.8)	
Paid occupation			0.150
Yes	115 (85.8)	19 (14.2)	
No	102 (79.1)	27 (20.9)	
Skin color/race			0.016
White/yellow	161 (79.7)	41 (20.3)	
Black/brown	95 (90.5)	10 (9.5)	
Per capita income, n=300			0.872
<BRL 156.00	8 (4.1)	1 (2.4)	
BRL 156.00 to BRL 456.00	73 (38.0)	16 (39.0)	
>BRL 456.00	111 (57.9)	24 (58.5)	
Previous pregnancies			0.065
Primiparous	88 (88.9)	11 (11.1)	
One	84 (84.8)	15 (15.1)	
Two or more	84 (77.1)	25 (22.9)	
Education (years of study), n=299			0.121
Up to 5	16 (88.9)	2 (11.1)	
6 to 9	26 (96.3)	1 (3.7)	
10 or more	213 (81.6)	48 (18.4)	
Marital coexistence, n=299			0.043
No partner	98 (89.1)	12 (10.9)	
With partner	157 (80.1)	39 (19.9)	
Planned pregnancy			0.422
Yes	100 (81.3)	23 (18.7)	
No	156 (84.8)	28 (15.2)	
Satisfaction with professionals, n=296			0.001
Very good	80 (72.1)	31 (28.0)	
Good	112 (88.2)	15 (11.8)	
Regular	48 (94.1)	3 (5.9)	
Bad	6 (100)	-	
Very bad	1 (100)	-	
Satisfaction with the team, n=300			<0.001
Very good	78 (69.6)	34 (30.4)	
Good	112 (91.7)	11 (8.9)	
Regular	30 (85.7)	5 (14.3)	
Bad	29 (96.7)	1 (3.3)	
Change team or UBS, n=299			0.002
Yes	65 (95.6)	3 (4.4)	
No	183 (79.2)	48 (20.8)	
Would recommend UBS, n=297			0.009
Yes	200 (80.3)	49 (19.7)	
No	46 (95.8)	2 (4.2)	

* Pearson’s χ^2^ test; ^†^ linear trend test; total of some variables different from n owing to missing data.

UBS: Basic Health Unit.

## DISCUSSION

This study sought to assess the quality of prenatal and postpartum care and its association with the sociodemographic characteristics of the population in a municipality in the metropolitan region of Curitiba, Paraná, Brazil. Most women did not receive adequate care, especially teenagers, women with black or brown skin, and those who did not live with a partner.

The criteria used in this study were multidimensional and covered different aspects of care. Similar studies agree that prenatal care has a low rate of adequacy. Research using National Program to Improve Access and Quality of Primary Care (PMAQ - *Programa Nacional de Melhoria do Acesso e da Qualidade da Atenção Básica* ) data found that 15% of users had adequate prenatal care.^( 20 )^ A study that used the assessment criteria proposed by the World Health Organization, with a list of 39 recommendations related to five types of intervention, did not find any pregnant women with prenatal care classified as adequate. An evaluation using criteria from the Prenatal and Birth Humanization Program (PHPN) found that <5% of prenatal care was considered adequate.^( 21 )^

Silva et al.^( 1 )^developed an evaluation model using an index called IPR/prenatal that combines data related to the structure, processes, and results based on PHPN indicators. Each item is classified as adequate or inappropriate, and based on the percentage of suitable items, prenatal care is classified as superior (100%), adequate (75%), intermediate (51–74%), or inadequate (<50%). The index classified 26.5% of prenatal care as adequate and 6.7% as superior to adequate.^( 1 )^

Among the clinical actions, the most commonly performed were measuring the uterine height and blood pressure, which was expected considering that these are the minimum data for evaluating pregnant women. However, less than 10% of women had dental appointments, which reduced the adequacy of the index.

Pregnancy is an opportunity to screen for cervical cancer, which is the fourth most common type of cancer related to mortality among women;^( 22 )^ however, this action was performed in less than one third of pregnant women. On the other hand, regarding educational activities, more than half of the women were provided guidance on the importance of exams. The prenatal protocol advises women not to miss the opportunity to undergo cervical cancer evaluation.^( 19 )^

The index that dealt with postpartum appointments had an adequacy rate of 13.3%. Within this index, second puerperal appointments were performed less frequently, verifying a pattern of deficiency in the monitoring of women at this stage. At the first appointment, the mother and newborn were evaluated to identify postpartum complications, considering that complications may arise at this stage leading to maternal and neonatal morbidity and mortality.^( 19 )^

The adequacy of prenatal care was associated with better satisfaction scores with the care received, with professionals and staff providing a greater adequacy of prenatal care, indicating that women who had a more positive perception of the service were more adherent to prenatal care. High levels of provider satisfaction involve bonding, guaranteeing unbureaucratic access to information and establishing good relationships between users and professionals.^( 2 , 19 )^

This study showed less adequacy of prenatal care among younger women, black or brown women, and those who did not live with a partner, which is consistent with the literature and reinforces the inequalities in prenatal care for the most vulnerable populations.^( 10 , 20 , 23 , 24 )^ Among the reasons for the late onset or low frequency of teenagers receiving prenatal care are late recognition of pregnancy, fear of family members, difficulty accepting pregnancy, difficulty accessing services, and a lack of knowledge about the importance of prenatal care.^( 24 , 25 )^

This work corroborates previous studies in which black or brown women received poorer quality prenatal care than women with white or yellow skin.^( 25 - 28 )^ These studies point out that these women had less access to labor analgesia, received less advice during prenatal care, and had fewer consultations and exams. Reports of obstetric violence were also more frequent in this group. These findings reinforce the need for institutional efforts to reduce inequalities in access to and quality of care based on skin color.

In prenatal care, this violence can appear in the form of a lack of access to the service, offer of a poor-quality service, poor structure to meet the demand for services, discrimination of users for any reason, or not ensuring that the pregnant woman enjoys all possibilities of assistance that she would be entitled to because of her clinical condition. On the other hand, prenatal care is a privileged space for combating obstetric violence because of the possibility of providing information that will make women aware of their rights and recognize the practice of violence.^( 27 )^ Women with partners had more adequate prenatal care than those without partners. The presence of a partner may be a factor that favors the woman’s attendance at prenatal care visits.^( 28 )^

The index applied in the research allowed for the identification of inequalities in the adequacy and differences between the care prescribed in the protocols and the reality experienced in everyday life and identify the greatest weaknesses. These data can help in the planning of management actions at the local level, such as training teams on the themes in which the lowest compliance rates were identified, identifying points for improvement, guiding discussions with the municipality’s service network, and verifying possible actions to confront and overcome the socioeconomic fragilities of women requiring assistance.

Access and quality must also be assessed by considering the coverage of health actions and services. Studies have shown that territories with better coverage rates have better health indicators considering that they promote the population’s access to services, which helps correct inequalities.^( 7 , 8 , 11 )^

It is worth noting that this study was conducted in the first year of the coronavirus 2019 pandemic. Access to prenatal care worsened during the pandemic period, which may have impacted the care for pregnant women. Therefore, the magnitude of this problem needs to be investigated further in future studies.^( 29 )^

One limitation of this study is the possibility of loss of information from pregnant women’s cards due to missing records. Secondly, the data that depended on recall about the care provided, such as participation in educational activities, may have been lacking because of recall bias.

Our evaluation indicates that prenatal care services are non-compliant with quality standards, suggesting that efforts should be made to improve all aspects of care. An important step is for health teams and municipal management to carry out systematic assessments to identify areas for improvement and dedicate efforts to change care practices.

## CONCLUSION

This study contributes to the evaluation of prenatal and postpartum care, which, by reaching higher quality standards, can help avoid complications by guaranteeing adequate interventions and timely treatment for women’s health conditions. Beyond establishing standards and procedures, quality prenatal care must include recognition of socioeconomic and cultural differences among users, as well as the prevention of violence.
